# *Staphylococcus aureus* Carriage in the Nasotracheal Cavities of White Stork Nestlings (*Ciconia ciconia*) in Spain: Genetic Diversity, Resistomes and Virulence Factors

**DOI:** 10.1007/s00248-023-02208-8

**Published:** 2023-03-24

**Authors:** Idris Nasir Abdullahi, Guillermo Juárez-Fernández, Ursula Höfle, Javier Latorre-Fernández, Teresa Cardona-Cabrera, David Mínguez-Romero, Myriam Zarazaga, Carmen Lozano, Carmen Torres

**Affiliations:** 1 Area Biochemistry and Molecular Biology, OneHealth-UR Research Group, University of La Rioja, 26006 Logroño, Spain; 2 SaBio (Health and Biotechnology) Research Group, Spanish Wildlife Research Institute IREC (CSIC-UCLM-JCCM), Ciudad Real, Spain

**Keywords:** *Staphylococcus aureus*, MSSA-CC398, Wild birds, MSSA-ST291, Antimicrobial resistance, White storks

## Abstract

The molecular ecology of *Staphylococcus aureus* in migratory birds (such as white storks) is necessary to understand their relevance in the “One Health” ecosystems. This study determined the nasotracheal carriage rates of *S. aureus* from white storks in Southern Spain and genetically characterized the within-host diversity. A collection of 67 *S. aureus* strains, previously obtained from 87 white stork nestlings (52 nasal and 85 tracheal samples) fed by their parents with food foraged in natural and landfill habitats, were tested for their antimicrobial resistance (AMR) phenotypes. Moreover, the AMR genotypes, immune evasion cluster (IEC), virulence genes and the detection of CC398 lineage were studied by PCR. The *spa* types and multilocus-sequencing-typing (MLST) were also determined by PCR and sequencing. *Staphylococcus aureus* carriage was found in 31% of storks (36.5%/11.9% in nasal/tracheal samples). All isolates were methicillin-susceptible (MSSA) and 8.8% of them were also susceptible to all tested antibiotics. The AMR phenotype/percentage/genes detected were as follows: penicillin/79.1%/*blaZ*; erythromycin-clindamycin-inducible/19.1%/*ermA, ermT*; tetracycline/11.9%/*tetK*; clindamycin/4.5%/*lnuA* and ciprofloxacin/4.5%. Twenty-one different *spa* types, including 2 new ones (t7778-ST15-CC15 and t18009-ST26-CC25), were detected and ascribed to 11 clonal complexes (CCs). MSSA-CC398 (8.2%), MSSA-CC15 (7.1%) and MSSA-ST291 (5.9%) were the most prevalent lineages in storks. Moreover, *tst*-positive (MSSA-CC22-t223 and MSSA-CC30-t1654), *eta-*positive (MSSA-CC9-t209) and *etb*-positive strains (MSSA-CC45-t015) were detected in four storks. The 18.5% of storks harboured distinct MSSA strains (with different lineages and/or AMR genes). Nestlings of storks foraging in landfills (10 CCs) had more diverse *S. aureus* strains than those of parents foraging in natural habitats (3 CCs). Low level of AMR was demonstrated among *S. aureus* strains. The predominance of MSSA-CC398 (an emergent clade) and toxigenic MSSA strains in stork nestlings highlight the need for continuous surveillance of *S. aureus* in wild birds.

**Supplementary Information:**

The online version contains supplementary material available at 10.1007/s00248-023-02208-8.

## Introduction

Humans are dependent on healthy ecosystems. The “One Health” approach, under which the World Health Organisation (WHO) has based the global strategy to tackle the problem of antimicrobial resistance (AMR), is based on the close link between human, animal and environmental health [1]. The recent focus on the “One Health” framework of research includes wild animals such as migratory birds [2].

Recently, white stork (*Ciconia ciconia*) among other wild birds (such as starlings, cowbirds and gulls) has attracted interest in the study of bacterial ecology and epidemiology as they have been shown to forage in human settlements, farmlands and dumpsites (which may include urban and hospital wastes) [3–5]. Also, during migration, storks can travel long distances across continents such as Africa to Europe and vice versa. These phenomena make storks potential reservoirs and carriers for transcontinental transmission of bacteria of public health concerns [2, 6].

The *Staphylococcus* genus is one of the common colonizers of the skin, nostrils and trachea of many wild animals [7–9]. Especially, *S. aureus* represents the most clinically important staphylococcal species in human and animal health [10]. Previously, our research group reported a high *S. aureus* carriage rate (34.8%) in white stork nestlings exposed to human residues [11]. Subsequently, it was shown that there was a 4.6% tracheal carriage of *S. aureus* among magpies and cinereous vultures [7]. Both studies included wild birds that have adapted to using human dumpsites/household residues as a major food source [7, 11]. Such foraging habits could lead to contamination or colonization of these wild birds by *S. aureus* [2].

Certain *S. aureus* strains have shown economic importance in livestock production and this fact is mainly represented by the emergence and spread of certain antimicrobial resistances and clones (livestock associated) that drastically reduce animal yield [12]. Methicillin-resistant *S. aureus* (MRSA) of clonal complex 398 (CC398) was first described in pigs and humans in contact with them in 2005 and was considered a livestock-associated (LA)-MRSA lineage [13, 14]; in the following years, MRSA-CC398 has been detected in different types of farm and wild animals, although the main host continues to be pigs and pig environment [2, 15]. Hence, MRSA-CC398 lineage is increasingly recognised as an occupational pathogen among pig industry workers such as farmers, slaughterhouse workers and veterinarians [16]. On the other hand, methicillin-susceptible *S. aureus-*CC398 (MSSA-CC398) is being detected as an emerging clone that has largely been implicated in human invasive infections in some countries, including Spain [17]. However, its genetic and epidemiological characteristics have not been fully elucidated in wild animals.

In a previous study, the nasotracheal microbiota of white stork nestlings of parents foraging in different habitats was analysed, obtaining over 800 bacteria isolates of different genera and species, including different staphylococcal species [18]. The present study is focused on characterizing the collection of *S. aureus* isolates obtained in that study, determining genetic lineage diversities, antimicrobial resistance mechanisms, immune evasion cluster (IEC) types and virulence factors of these isolates. Consequently, we analysed and compared *S. aureus* isolates obtained from nasal and tracheal samples of white stork nestlings from nests associated to either natural habitat and landfills and that are thus fed by their parents with differentially landfill or natural habitat foraged food, in Southern Spain.

## Materials and Methods

### Sample Analysis and *S. aureus* Recovery

In July 2021, nasal and tracheal swab samples of 87 white stork nestlings were collected during ringing activities and accounted for a total of 137 samples: 85 tracheal (T) and 52 nasal (N). Of these animals, both nasal and tracheal samples were collected from 49 of them. The storks corresponded to four different colonies of regions of southern Spain with different foraging strategies. Storks in colonies 1 and 2 were located in natural habitats in open woodland devoted to extensive livestock grazing where storks forage, while those in colonies 3 and 4 are located on or close to landfills and the adults forage in landfills. These samples were processed for the recovery of the bacterial community diversities, which included *S. aureus* [18]. Potential *S. aureus* colonies (up to 6 per sample) were randomly selected based on colour, size and/or hemolysis. The strains were identified by matrix-assisted laser desorption/ionisation time-of-flight (MALDI-TOF; Bruker Daltonics, Bremen, Germany) using the standard extraction protocol recommended by Bruker Daltonics as previously described [19]. The strains identified as *S. aureus* (*n* = 67) were obtained from 29 of the 137 samples (21.2%) of 27 of the 87 storks (31%) analysed in a previous study [18]. Specifically, the prevalence of *S. aureus* carriage was higher in the nasal than in the tracheal cavities (36.5% and 11.9%, respectively), as indicated in the previous study [18]. In relation to the foraging habits of the parents of the storks, 35.0% and 37.5% nasal *S. aureus* carriage was found in stork nestlings whose parents foraged in natural habitats and landfills, respectively. Moreover, 7.1% and 16.3% tracheal carriage was found in stork nestlings fed natural habitat and landfill foraged food, respectively (Supplementary Table S1) [18]. All these 67 *S. aureus* strains were further characterized in the present study.

### *Staphylococcus aureus* DNA Extraction

For DNA extraction, the strains were streaked on BHI agar and incubated for 24 h at 37 °C. An isolated colony was suspended in 45 μL of sterile MiliQ water and later 5 μL of lysostaphin was added (1 mg/mL) (Sigma®). The mixture was vortexed and incubated for 10 min at 37 °C. Forty-five microliters of sterile MiliQ water, 150 μL of Tris–HCl (0.1 M, pH 8) and 5 μL of proteinase K (2 mg/mL) (Sigma®) were added. This was vortexed and incubated for 10 min at 60 °C. Finally, it was boiled for 5 min at 100 °C and centrifuged at 12,000 revolutions per minute for 3 min. The DNA was stored at − 20 °C.

### Antimicrobial Susceptibility Testing and Detection of Resistance Genes

Susceptibility testing for 13 antimicrobial agents was performed by the disc diffusion method with all *S. aureus* strains following the recommendations and breakpoints of the European Committee on Antimicrobial Susceptibility Testing [20]. The antimicrobial agents tested were as follows (μg/disc): penicillin (1 unit), cefoxitin (30), erythromycin (15), clindamycin (2), gentamicin (10), tobramycin (10), tetracycline (30), ciprofloxacin (5), chloramphenicol (30), linezolid (10), mupirocin (200) and trimethoprim-sulfamethoxazole (1.25 + 23.75).

The presence of the following resistance genes was tested by single PCRs, selected according to the antimicrobial resistance phenotype: beta-lactams (*blaZ*, *mecA* and *mecC*), erythromycin and clindamycin (*ermA*, *ermB*, *ermC*, *ermT*, *lnuA* and *lnuB*) and tetracycline (*tetL*, *tetM* and *tetK*). Primers and conditions of PCRs for the AMR genes tested are included in Supplementary Table S2.

### Detection of Virulence Encoding Genes

The presence of the virulence genes *tst*, *lukS-PV/lukF-PV*, *eta* and *etb* (encoding the toxin of toxic shock syndrome, Panton-Valentine leucocidin and exfoliative toxins A and B, respectively) were tested by PCR in every strain. Also, the immune evasion cluster (IEC) genes (*scn*, *chp*, *sak*, *sea* and *sep*) were analysed and classified accordingly into seven different IEC types (A–G), based on the combination of the positive genes. The *scn* gene (encoding the staphylococcal complement inhibitor) was used as a marker of the IEC system. Primers and conditions for all PCRs for virulence and IEC genes are included in Supplementary Table S2.

### Genetic Characterization

All *S. aureus* strains were characterized for *spa* genotyping by PCR/Sanger sequencing. New repeat combinations were submitted to the Ridom *spa* Server (https://spa.ridom.de/submission.shtml). CC398 clone was determined by a specific PCR protocol for the *sau1*-*hsdS1* variant developed by Stegger et al. [21]. Primers and conditions are included in Supplementary Table S2. The clonal complex (CC) of the strains was assigned, when possible, according to the *spa* types.

Multilocus sequence typing (MLST) was performed in selected *S. aureus* (strains with *spa* types that were detected in many animals or newly detected *spa* types). The 7 housekeeping genes (*arcC*, *aroE*, *glpF*, *gmk*, *pta*, *tpi* and *yqiL*) of *S. aureus* strains were amplified as previously described (Supplementary Table S2), and sequence type (ST) was assigned from sequence analyses on the MLST database (http://pubmlst.org/).

Positive controls from the collection of the Universidad de La Rioja were included in all the PCR assays in this study.

### Statistical Analysis

Statistical Package for Social Sciences (SPSS) Version 26 (IBM, California, USA) was used for data analysis. Associations between categorical variables (such as type of samples and prevalence of bacterial genus and species) were compared using the chi-square test. Data were subjected to univariate logistic regression to compute odds ratio (OR) at a 95% confidence interval (95% CI) between the prevalence rate of *S. aureus* and the foraging habit of the colonized and non-colonized storks. These tests were carried out as two-tailed and outcomes with a probability value less than 0.05 were considered statistically significant.

## Results

### Antimicrobial Resistance Phenotypes and Genotypes and Virulence Determinants among Nasotracheal *S. aureus* Strains from Stork Nestlings

**Fig. 1:**
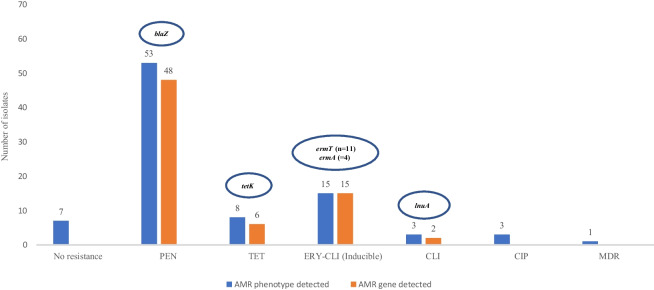
Antimicrobial resistance phenotypes and genotypes in the 67 *S. aureus* isolates recovered from white stork nestlings. Abbreviation: CLI clindamycin, CIP ciprofloxacin, ERY erythromycin, MDR multidrug resistance, PEN penicillin; TET tetracycline

All the 67 *S. aureus* strains recovered of nasal and tracheal samples of white stork nestlings were MSSA, and 7 of them (8.8%) were susceptible to all the antibiotics tested (Fig. 1). The most frequently identified AMR was to penicillin (PEN^R^) (*n* = 53, 79.1%) with *blaZ* detected in 90.6% of PEN^R^ strains. Other AMR-phenotypes/percentage/genes detected were as follows: erythromycin + clindamycin-inducible/19.1%/*ermA*, *ermT*; tetracycline/11.9%/*tetK*; clindamycin/4.5%/*lnuA* and ciprofloxacin/4.5%. Multidrug resistance (resistance to at least 3 different families of antibiotics) was identified in one strain (1.5%) (Fig. 1). None of the 67 strains tested carried the *luk-S/F-PV* gene but the *tst*, *eta* and *etb* genes was detected in strains of 4 nestling storks.

### Genetic Lineages Versus Antimicrobial Resistance Phenotypes/Genotypes and Virulence Content

Of all the 67 MSSA strains, 21 different *spa* types were detected including 2 new *spa* types (t7778-ST15-CC15 and t18009-ST26-CC25), and they were ascribed to 11 clonal complexes (CCs): CC1, CC5, CC9, CC15, CC22, CC25, CC30, CC45, CC97, CC130 and CC398 (Table 1). Twelve of the MSSA strains (from 7 storks), belonged to CC398 lineage and *spa* types t571 (*n* = 5), t1451 (*n* = 3) and t1456 (*n* = 4); all of them were of the IEC-type C. Six of these CC398 strains were penicillin-resistant and presented the erythromycin-clindamycin inducible resistance phenotype with the corresponding genes *blaZ* and *ermT*. Moreover, two of the MSSA-CC398 strains were ciprofloxacin-resistant (Table 1). In addition, 11 strains obtained from six storks corresponded to the lineage MSSA-ST291-t2313/IEC type B, being ST291 a double-locus variant of ST398. Putting together, 14.1% of storks carried MSSA-CC398 or MRSA-ST291 (closely related lineages). Also, two MSSA-CC130 (*spa*-type t6220) strains were identified, and they were susceptible to all antimicrobial agents. Other genetic lineages detected in high number were the following ones (number of strains): (a) MSSA-CC5-t227/IEC-B (*n* = 6) and CC5-t1094/IEC negative (*n* = 1); (b) MSSA-CC15-t774/IEC type-E (*n* = 5); (c) MSSA-CC15-t085/t335/IEC-C (*n* = 5); (d) MSSA-CC9-t209/IEC negative (*n* = 4) and (e) MSSA-CC45-t015/IEC-B (*n* = 3). All genetic lineages and their corresponding AMR phenotypes are presented in Table 1. One *S. aureus* strain showed a multidrug-resistant (MDR) phenotype (PEN-ERY-CLI^I^-CIP) and was typed as MSSA-CC398-t571. Interestingly, two of the storks carried *tst*-positive *S. aureus* strains of different lineages (MSSA-CC22-t223-IEC-B and MSSA-CC30-t1654-IEC-negative, both of them PEN^R^). Also, *eta*-positive (MSSA-CC9-t209-*scn* negative) and *etb*-positive strains (MSSA-CC45-t015-IEC-B) were detected in two additional storks. However, all strains were *luk-F/S-PV*-negative (Table 1).

**Table 1:** Antimicrobial resistance Phenotype and Genotype of the 67 MSSA isolates in relation to their *spa*-type and clonal complexes

*spa*-type	Clonal complex (CC)^a^	N^o^ of isolates**/**N^o^ of storks	Virulence genes detected^b^	Immune evasion cluster (IEC) type (number of isolates)	AMR phenotypes^c^	AMR genes^c^
t571	CC398	5/3	ND	C (5)	PEN^3^-CIP^2^-ERY^4^-CLI^ind4^	*blaZ*^1^, *ermT*^4^
t1451	CC398	3/2	ND	C (3)	PEN^1^-ERY^3^-CLI^ind3^	*ermT*^3^
t1456	CC398	4/2	ND	C (4)	PEN^2^-ERY^4^-CLI^ind4^	*blaZ*^2^, *ermT*^4^
t127	CC1	3/2	ND	Negative (3)	CLI^3^-TET^3^	*tetK*^3^, *lnuA*^3^
t227	CC5	6/2	ND	B (6)	PEN^6^-CLI^1^	*blaZ*^6^
t1094	CC5	1/1	ND	Negative	PEN^1^-CIP^1^	*blaZ*^1^
t209	CC9	4/1	*eta*^4+^	Negative	PEN^4^-ERY^4^-CLI^4^	*blaZ*^4^, *ermA*^4^
t085	CC15	5/2	ND	C (5)	PEN^5^	*blaZ*^5^
t774	CC15	5/3	ND	E (5)	PEN^5^-TET^5^	*blaZ*^4^, *tetK*^4^
t7778^d^	CC15/ST15	4/2	ND	C (1), negative (3)	PEN^3^	*blaZ*^3^
t223 t18009^d^ t1654	CC22 CC25/ST26 CC30	2/1 3/1 1/1	*tst*^2+^ ND *tst*^1+^	B (2) B (3) Negative	PEN^2^ PEN^3^ PEN	*blaZ*-negative *blaZ*^3^ *blaZ*^1^
t015	CC45	3/1	*etb*^3+^	B (3)	PEN^3^	*blaZ*^3^
t521	CC97	2/1	ND	E (2)	Susceptible	NT^f^
t3380	CC97	3/2	ND	E (3)	PEN^1^	*blaZ*^1^
t6220	CC130	2/1	ND	Negative (2)	Susceptible	NT^f^
t2313	ST291^e^	11/5	ND	B (12)	PEN^11^	*blaZ*^12^

Abbreviation: *CLI*^*ind*^ clindamycin inducible, *CLI* clindamycin, *CIP* ciprofloxacin, *ERY* erythromycin, *PEN* penicillin, *TET* tetracycline

^a^CC assigned according to the *spa-*type, except for CC398 (determined by specific PCR) and CC25/ST26, CC15/ST15 and ST291 (determined by MLST)

^b^ All *tst-*, *eta-*, *etb-*positive strains were confirmed by sequencing

^c^In superscript is the number of isolates that presented the specific phenotype/genotype of AMR

^d^New *spa* types

^e^ST291 is a double-locus variant of ST398

^f^*NT *Not tested

*ND* Not detected (negative for *lukS-PV/lukF-PV*, *tst*, *eta*, *etb*)

Stork nestlings whose parents foraged in landfills presented relatively more genetically diverse *S. aureus* strains (10 CCs and lineage ST291) than those of parents foraging in natural habitats (only 3 CCs and lineage ST291) (Table 2). Moreover, all *tst*-, *eta*- and *etb*-positive strains were recovered of nestlings whose parents foraged in landfills.

**Table 2:** Genetic lineages variation of *S. aureus* isolates from white stork nestlings according to the foraging habits of their parents

Genetic lineage	Nestlings of parent storks foraging in natural habitat		Nestlings of parent storks foraging in landfills	
	*spa* types (number) in nasal samples	*spa* types (number) in tracheal samples	*spa* types (number) in nasal samples	*spa* types (number) in tracheal samples
CC1	ND	ND	t127 (3)	ND
CC5	ND	ND	t1094 (1)	t227 (6)
CC9	ND	ND	ND	t209 (4)
CC30	ND	ND	t1654 (1)	ND
CC15	ND	ND	t085 (4), t774 (1), t335 (1)	t774 (4)
CC15/ST15	ND	ND	t085 (1), t7778 (2)	t7778 (2)
CC22 CC25/ST26	ND ND	ND ND	t309 (2) ND	ND t18009 (3)
CC45	ND	ND	t015 (3)	ND
CC97	t3380 (1)	t3380 (1)	t521 (2), t3380 (1)	ND
CC130	ND	t6220 (2)	ND	ND
CC398	t1456 (3)	t571 (1), t1456 (1)	t571 (4), t1451 (3)	ND
ST291 Total	t2313 (4) 8	t2313 (1) 5	t2313 (5) 33	t2313 (2) 21

*ND *Not detected

**Fig. 2:**
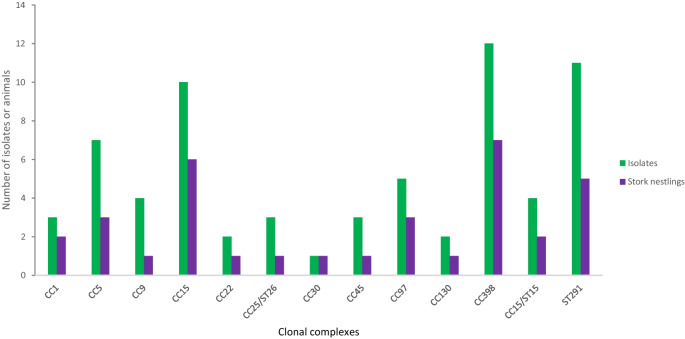
Number of isolates and white stork nestlings in which the different *S. aureus* clonal complexes were detected

Of the 12 MSSA-CC398 strains obtained from seven positive storks (Fig. 2), five strains were from nestlings of parents that forage in natural habitat while the other seven were obtained from stork nestlings of parents foraging in landfills (Table 2). Aside from MSSA-CC398 strains that were found in high frequency, 11 MSSA-ST291-*spa*-type t2313 strains were also found in 5 storks. Others include MSSA-CC15, MSSA-CC5 and CC97 in 5, 3 and 3 storks, respectively. However, MSSA of the CC9, CC22, CC25, CC30, CC45 and CC130 lineages were all found in one stork each (Fig. 2).

### Intra-sample Variation of Genetic Lineages or AMR Genotypes of *S. aureus* Strains from Stork Nestlings

Five of 27 storks positive for *S. aureus* (18.5%) harboured *S. aureus* strains with diverse *spa*-types or AMR genotypes in the same animal. Between 2 to 5 genetically distinct *S. aureus* strains were detected in these animals (Table 3). Two storks had genetic lineage variation and AMR phenotypes/genotypes in the tracheal *S. aureus* carriages: (1) MSSA-ST291-t2313*-*PEN^R^-*blaZ* and MSSA-CC130*-*t6620-PEN^S^ and (2) two MSSA-CC15-t774 and one MSSA-t7778 strain (Table 3). Moreover, a stork with nasal carriage of MSSA-CC398 had 2 distinct *spa* types (t571 and t1451): one of the MSSA-CC398 strains was completely susceptible to antibiotics, whereas the other carried the *ermT* gene (Table 3).

**Table 3:** Intra-sample and intra-host variation of genetic lineages or AMR genotypes of *S. aureus* isolates

Stork’s ID	Sample type	Number of isolates	IEC type	AMR phenotypes (gene detected)	*spa*-type/CC
436	Tracheal	1	B	PEN (*blaZ*)	t2313
	Tracheal	2	Negative	Susceptible	t6620/CC130
489	Nasal	1	C	PEN (*blaZ*)	t7778/ST15/CC15
	Nasal	1	Negative	PEN (*blaZ*)	t1094/CC5
	Nasal	1	C	Susceptible	t7778/ST15/CC15
490	Tracheal	2	Negative	PEN (*blaZ*)	t7778/ST15/CC15
	Tracheal	1	E	PEN (*blaZ*)	t774/CC15
505	Nasal	1	C	PEN-ERY-CLI^ind^ (*ermT*)	t571/ CC398
	Nasal	2	C	PEN-ERY-CLI^ind^ (*ermT*)	t1451/CC398
	Nasal	1	C	Susceptible	t571/CC398
546	Nasal	3	Negative	CLI^3^-TET^3^ (*tetK*^3^, *inuA*^3^)	t127/CC1
	Nasal	1	E	Susceptible	t3380/ CC97
	Nasal Tracheal	1 3	E B (3)	PEN-TET (*blaZ*, *tetK*) PEN (*blaZ*)	t774/CC15 t227/CC5

Abbrev. *CLI* clindamycin, *CIP* ciprofloxacin, *ERY* erythromycin, *PEN* penicillin, *TET* tetracycline, *IEC* immune evasion cluster, *AMR* antimicrobial resistance

## Discussion

The contemporary recommendations on the molecular epidemiology of *S. aureus* using the “One health” approach have recognised the inclusion of wild animals and other environmental reservoirs. It is important to study not only *S. aureus* prevalence, CCs and AMR, but to also search for their diversity and variance along with host ecological factors such as sampling location and host foraging behaviours.

*Staphylococcus aureus* nasotracheal carriage rate of 31% was found in the white stork nestlings analysed in this study, with higher prevalence in nasal samples (36.5%) than in traquel samples (11.9%) of the nestlings [18]. Other studies analysed the tracheal *S. aureu*s carriage rate in wild birds with varios frequencies (4.6–34.8%) [7, 11, 22, 23]. 

In relation to the foraging habit of the birds, nestling storks of parents that forage in landfills had a relatively higher *S. aureus* carriage rate than those with parents that forage in natural habitats [18]. This could be because storks foraging in natural habitats might also have relatively lesser chances of *S. aureus* contamination than those foraging in landfills [7]. In the study of Gómez et al. [11], the high *S. aureus* carriage could be attributed to the stork nestling’s exposure to human residues. Thus, the exploitation of human household residues as a major food source seems to be a risk factor for the acquisition of *S. aureus* by wild birds [2]. Especially in the case of migratory birds (such as storks), these could then act as vectors between habitats, regions and even continents [6].

For more than a decade, MRSA-CC398 has consistently been detected in humans who had contact with livestock (mainly pigs and piggery environments). However, lately, the MSSA-CC398 strains have also attracted interest for epidemiological and evolutionary purposes because MSSA-CC398 strains could be implicated in emergent invasive human infections [17]; many of the MSSA-CC398 strains seem to correspond to the livestock-independent clade, predominantly IEC-positive, with the *spa* t571 as predominant in the human infections [17]. Nevertheless, a major microecological concern is that this clade has also been detected in other non-human environments. In this study, stork nestlings were frequent carriers of MSSA-CC398, the assumed “human clade”. This opens questions about its diverse origin especially, as the MSSA-CC398 was found in both stork nestlings fed food foraged from natural habitats and landfills.

In this study, it appears that storks, especially those fed food foraged in landfills, had a higher MSSA-CC398 carriage rate. Potentially, some of the nestlings in this study were fed parts of animal carcasses and discarded household residue from refuse plants that could be colonized by certain human-associated *S. aureus* clones (such as the MSSA-CC398). This could be one of the reasons why certain wild birds often carry MSSA-CC398 clones mainly adapted to humans [2]. In our study, all MSSA-CC398 strains presented the IEC type C, which is the most frequently found in MSSA-CC398 [17]. Resistance to erythromycin-clindamycin inducible mediated by the *ermT* gene was identified in > 90% of our MSSA-CC398 strains. Thus, the *ermT* gene and IEC type C seem to be clear genetic markers of the MSSA-CC398 clone. However, some strains without these genes have previously been reported [17].

Recently, an increased detection of penicillin susceptibility phenotype (PEN^S^) among invasive MSSA human strains has been observed, opening therapeutic opportunities for these infections; this phenotype has frequently been found among *scn*-negative or CC398 strains, which suggests a potential animal-associated link [17]. In our study, 20.9% of the *S. aureus* strains were PEN^S^. Aside from the MSSA-CC398-PEN^S^ clone with a high potential for invasive infections, other MSSA-PEN^S^ clones, such as the CC5, were often reported to cause bloodstream infections (BSIs) in a large-scale study in Spain [17]. The lineage CC5 has also been associated with avian strains and it is thought that a subtype of human CC5 was implicated in a human-to-poultry host jump [24]. Interestingly, 6 out of 7 of our MSSA-CC5 strains harboured IEC genes (IEC type B).

Another relevant CC detected was CC130, which was identified in two MSSA strains. The MSSA-CC130 is related to small ruminants (such as sheep) and it has been proposed that this clonal lineage evolved from humans to ruminants [25]. None of the CC130 strains contained IEC genes. This finding is consistent with a previous study that indicated that CC130-MSSA appears to be a common lineage in sheep causing several infections [26]. In relation to the MSSA-CC130 from our study, the two strains were from nestlings of parent storks foraging in natural habitats, that consists of open woodland employed for extensive livestock grazing that involves mainly ruminants (sheep and cattle).

Besides the MSSA-CC398 lineage, MSSA-CC15 and MSSA-ST291 were detected in high frequency from both nasal and tracheal samples. The MSSA-ST291 lineage has been reported by most previous studies, and is globally distributed [27–32]. It is important to remark that ST291 is a double-locus variant of ST398 and has previously been misassigned to CC398 [30, 31]. Although scarcely described in recent times, MSSA-ST291-*spa-*type t2313 has been responsible for invasive infections with low-level AMR (often carrying only *blaZ* gene). However, it has previously been detected in healthy people in Germany [32].

The pathogenicity of MSSA is largely determined by the presence of certain important virulence factors (especially Panton-Valentine leucocidin, toxic shock syndrome toxin and exfoliative toxins). To our knowledge, this is the first time that *tst*-carrying MSSA strains of the genetic lineage MSSA-CC22 has been found in storks. Nevertheless, *tst*-carrying MSSA-CC30-t012 strains were previously reported by Gomez et al. [10] in storks. Also, *tst*-positive MSSA-CC522 strains were previously reported in other wild animals (such as wild boar) in Spain as well in healthy ewe in Tunisia [33, 34]. It is worth mentioning that *tst*-carrying MSSA-CC22 strains (of *spa*-type t790) have been associated to wound infections in Iran [35]. A possible link between the detection of MSSA strains containing virulence genes in wild birds and livestock could be that the storks foraged on pastures or in farm areas contaminated with livestock droppings. Also, the toxigenic MSSA from storks in the present study originated all of them from nestlings fed food foraged in landfills which might contain visceral remainings including intestines. In addition to the presence of *tst* gene, the detection of MSSA-*eta-* and *etb-*positive strains in the two storks from our study are also relevant and can pose clinical and public health implications. We are not aware of any previous report of *etb*-positive MSSA in wild birds.

In this study, we report two new *spa* types. One of them, t7778 belonged to the ST15/CC15 while the other t18009 belonged to the ST26/CC25 lineage. Concerning the MSSA-CC15, this lineage has previously been described with diverse *spa* types to be circulating in many European countries and Asia [36–39]. Concerning the MSSA-ST26, this lineage has rarely been reported in previous studies. However, worthy to mention is the detection of MSSA-ST26-*blaZ*-positive strains from human oral samples in Japan [40]. Hence, these CCs need to be monitored to fully understand their evolution in the One Health ecosystem.

The abundance and diversity of genetic lineages and antimicrobial resistance genes (ARG), within and between samples (nasal and tracheal) of healthy stork nestlings are of public health concern. In all, there are significantly more distinct CCs and ARGs in nasal than tracheal cavities, and stork nestlings fed food foraged in landfills compared to those fed food foraged in natural habitats. There are several potential reasons for these findings. Notably, many sites in the nasal cavity host highly complex and robust microbial biofilm structures. It has been posited that the compact structure of microbes within nasotracheal epithelial provides a favourable environment for the acquisition of ARGs [41, 42].

For the stork nestlings whose parents forage in landfills, it is not surprising that the *S. aureus* strains were highly diverse as landfills are often “dumping sites” of all sorts of solid wastes that act as complex microbiological niche favouring and giving rise to an abundant microbial community with potentially greater numbers of distinct strains and ARGs [42, 43]. This highlights the importance of characterizing the resistomes across different body sites of potential wildlife hosts to uncover the AMR carriage and transmission potential in wild animals.

## Conclusion

One-third of the nestling storks of this study were *S. aureus* carriers in the nasal and/or tracheal cavity, being *S. aureus* more frequently found in nasal samples. All *S. aureus* recovered were MSSA, showing in most cases low AMR levels. About 8% of the storks carried MSSA-CC398 strains, harbouring mainly *ermT* gene and the IEC-type-C. Remarkably, intra-sample and intra-animal variations of genetic lineages and AMR patterns were identified. Moreover, MSSA-CC398 clade found in these migratory birds suggests they may have a major role in transmission across various sources and the environment. The detection of *tst-, eta- and etb-*carrying *S. aureus* strains is of great public health concern. Considering the genetic diversity of the *S. aureus* strains, more effective control measures are needed to control the dissemination of *S. aureus* across the “One Health” ecosystems.

## Supplementary Information

Below is the link to the electronic supplementary material.

Supplementary file1 (DOCX 21 KB)

Supplementary file2 (DOCX 24 KB)

## Data Availability

The data generated from this study can be available on request through the corresponding author (Carmen Torres).
