# Supplementary material for: Staphylococcus aureus Carriage in the Nasotracheal Cavities of White Stork Nestlings (Ciconia ciconia) in Spain: Genetic Diversity, Resistomes and Virulence Factors
Source: Microb Ecol. 2023 Mar 24;86(3):1993–2002. doi: 10.1007/s00248-023-02208-8 (PMC10497646; doi:10.1007/s00248-023-02208-8)
Supplement: Supplementary file 1 — Supplementary file1 (DOCX 21 KB) [file 248_2023_2208_MOESM1_ESM.docx]

**Table S1.** Prevalence pattern and association of *S. aureus* in white stork nestlings with the foraging habits of their parents^a^

| Sample source | N^o^ of samples (or animals) tested | N^o^ of samples (or white storks) with *S. aureus* | N^o^ of nestlings with parent storks feeding in natural areas* / N^o^ with *S. aureus* (%) | N^o^ of nestlings with parent storks feeding in landfills/ N^o^ with *S. aureus* (%) | OR (95% CI) | *p* value |
| --- | --- | --- | --- | --- | --- | --- |
| Nasal | 52 | 19 | 20/7 (35.0) | 32/12 (37.5) | 1.26 (0.39-4.03) | 0.69 |
| Tracheal | 85 | 10 | 42/3 (7.1) | 43/7 (16.3) | 2.39 (0.92- 6.25) | 0.07 |
| Paired nasal and tracheal | 49 | 19 | 18/6 (33.3) | 31/13 (41.9) | 1.44 (0.95- 6.34) | 0.55 |
| Either nasal or tracheal | 87 | 26 | 44/9 (20.4) | 43/17 (39.5) | 2.45 (0.95- 6.34) | 0.09 |

Analysis by bivariate logistic analysis at 95% CI

* Referent group

^a^Data obtained from a previous study [18]
