# Supplementary material for: Staphylococcus aureus Carriage in the Nasotracheal Cavities of White Stork Nestlings (Ciconia ciconia) in Spain: Genetic Diversity, Resistomes and Virulence Factors
Source: Microb Ecol. 2023 Mar 24;86(3):1993–2002. doi: 10.1007/s00248-023-02208-8 (PMC10497646; doi:10.1007/s00248-023-02208-8)
Supplement: Supplementary file 2 — Supplementary file2 (DOCX 24 KB) [file 248_2023_2208_MOESM2_ESM.docx]

**Table S2.** Primers and conditions of the PCR performed in this study.

| **Gene** | **Primers’ oligonucleotide (5’**⇒**3’)** | **Amplicon size** | **Reference** |
| --- | --- | --- | --- |
| *blaZ* | F: CAGTTCACATGCCAAAGAG | 772 bp | [1] |
|  | R: TACACTCTTGGCGGTTTC |  |  |
| *mecA* | F: GGGATCATAGCGTCATTATTC | 527 bp | [2] |
|  | R: AACGATTGTGACACGATAGCC |  |  |
| *mecC* | F: GCTCCTAATGCTAATGCA | 304 bp | [3] |
|  | R: TAAGCAATAATGACTACC |  |  |
| *ermA* | F: TCTAAAAAGCATGTAAAAGAA | 645 bp | [4] |
|  | R: CTTCGATAGTTTATTAATATTAG |  |  |
| *ermB* | F: GAAAAGTACTCAACCAAATA | 639 bp | [4] |
|  | R: AGTAACGGTACTTAAATTGTTTA |  |  |
| *ermC* | F: TCAAAACATAATATAGATAAA | 642 bp | [4] |
|  | R: GCTAATATTGTTTAAATCGTCAAT |  |  |
| *ermT* | F: CCGCCATTGAAATAGATCCT | 200 bp | [5] |
|  | R: TTCTGTAGCTGTGCTTTCAAAAA |  |  |
|  | R: ATCATGTGATGTAAACAAAAT |  |  |
| *lnuA* | F: GGTGGCTGGGGGGTAGATGTATTAACTGG | 323 bp | [6] |
|  | R: GCTTCTTTTGAAATACATGGTATTTTTCGATC |  |  |
| *lnuB* | F: CCTACCTATTGTTTGTGGAA | 499 bp | [7] |
|  | R: ATAACGTTACTCTCCTATTC |  |  |
|  | R: TGGCACAGATGGTCATAACC |  |  |
| *tetL* | F: CATTTGGTCTTATTGGATCG | 456 bp | [8] |
|  | R: ATTACACTTCCGATTTCGG |  |  |
| *tetM* | F: GTTAAATAGTGTTCTTGGAG | 576 bp | [8] |
|  | R: CTAAGATATGGCTCTAACAA |  |  |
| *tetK* | F: TTAGGTGAAGGGTTAGGTCC | 697 bp | [8] |
|  | R: GCAAACTCATTCCAGAAGCA |  |  |
| *scn* | F: AGCACAAGCTTGCCAACATCG | 257 bp | [9] |
|  | R: TTAATATTTACTTTTTAGTGC |  |  |
| *chp* | F: TTTACTTTTGAACCGTTTCCTAC | 366 bp | [9] |
|  | R: CGTCCTGAATTCTTAGTATGCATATTCATTAG |  |  |
| *sak* | F: AAGGCGATGACGCGAGTTAT | 223 bp | [9] |
|  | R: GCGCTTGGATCTAATTCAAC |  |  |
| *sea* | F: AGATCATTCGTGGTATAACG | 344 bp | [9] |
|  | R: TTAACCGAAGGTTCTGTAGA |  |  |
| *sep* | F: AATCATAACCAACCGAATCA | 196 bp | [9] |
|  | R: TCATAATGGAAGTGCTATAA |  |  |
|  | R: CCAGATCTATCTTCTGATTCAGC |  |  |
| *tst* | F: TTCACTATTTGTAAAAGTGTCAGACCCACT | 180 bp | [10] |
|  | R: TACTAATGAATTTTTTTATCGTAAGCCCTT |  |  |
| *lukS-PV/lukF-PV* | F: ATCATTAGGTAAAATGTCTGGACATGATCCA | 443 bp | [6] |
|  | R: GCATCAAGTGTATTGGATAGCAAAAGC |  |  |
| *eta* | F: ACTGTAGGAGCTAGTGCATTTGT | 190 bp | [11] |
|  | R: TGGATACTTTTGTCTATCTTTTTCATCAAC |  |  |
| *etb* | F: CAGATAAAGAGCTTTATACACACATTAC | 612 bp | [11] |
|  | R: AGTGAACTTATCTTTCTATTGAAAAACACTC |  |  |
| *arcC* | F: TTGATTCACCAGCGCGTATTGTC | 456 pb | [12] |
|  | R: AGGTATCTGCTTCAATCAGCG |  |  |
| *aroE* | F: ATCGGAAATCCTATTTCACATTC | 456 pb | [12] |
|  | R: GGTGTTGTATTAATAACGATATC |  |  |
| *glpF* | F: CTAGGAACTGCAATCTTAATCC | 465 pb | [12] |
|  | R: TGGTAAAATCGCATGTCCAATTC |  |  |
| *gmk* | F: ATCGTTTTATCGGGACCATC | 429 bp | [12] |
|  | R: TCATTAACTACAACGTAATCGTA |  |  |
| *pta* | F: GTTAAAATCGTATTACCTGAAGG | 474 bp | [12] |
|  | R: TTTGCACCTTCTAACAATTGTAC |  |  |
| *tpi* | F: TCGTTCATTCTGAACGTCGTGAA | 402 bp | [12] |
|  | R: GAACCATTCGCCCCATGAA |  |  |
| *yqiL* | F: CAGCATACAGGACACCTATTGGC | 516 bp | [12] |
|  | R: CGTTGAGGAATCGATACTGGAAC |  |  |
| *sau1-hsdS1* | F: AGGGTTTGAAGGCGAATGGG | 296 bp | [13] |
|  | R: CAGTATAAAGAGGTGACATGACCCCT |  |  |

1. Schnellmann C, Gerber V, Rossano A, Jaquier V, Panchaud Y, Doherr MG, Thomann A, Straub R, Perreten V (2006) Presence of new *mecA* and *mph*(C) variants conferring antibiotic resistance in *Staphylococcus* spp. isolated from the skin of horses before and after clinic admission. J Clin Microbiol. 44, 4444–4454.
2. Poulsen AB, Skov R, Pallesen LV (2003) Detection of methicillin resistance in coagulase-negative staphylococci and in staphylococci directly from simulated blood cultures using the EVIGENE MRSA Detection Kit. J Antimicrob Chemother 51, 419–421.
3. Cuny C, Layer F, Strommenger B, Witte W (2011) Rare occurrence of methicillin -resistant *Staphylococcus aureus* CC130 with a novel *mecA* homologue in humans in Germany PLoS One 6.
4. Sutcliffe J, Grebe T, Tait-Kamradt A, Wondrack L (1996) Detection of erythromycin-resistant determinants by PCR. Antimicrob Agents Chemother 40, 2562–2566.
5. Gómez-Sanz E, Torres C, Lozano C, Fernández-Pérez R, Aspiroz C, Ruiz-Larrea F, Zarazaga M (2010) Detection, molecular characterization, and clonal diversity of methicillin-resistant Staphylococcus aureus CC398 and CC97 in Spanish slaughter pigs of different age groups. Foodborne Pathog Dis; 7(10):1269-77
6. Lina G, Quaglia A, Reverdy ME, Leclercq R, Vandenesch F, Etienne J (1999) Distribution of genes encoding resistance to macrolides, lincosamides, and streptogramins among staphylococci. Antimicrob. Agents Chemother; 43, 1062–1066.
7. Bozdogan B, Berrezouga L, Kou MS, Yurek DA, Farley KA, Stockman BJ, Leclercq R (1999) A new resistance gene, *linB*, conferring resistance to lincosamides by nucleotidylation in *Enterococcus faecium* HM1025. Antimicrob Agents Chemother; 43, 925–929.
8. Aarestrup FM, Agerso Y, Gerner-Smidt P, Madsen M, Jensen LB (2000) Comparison of antimicrobial resistance phenotypes and resistance genes in *Enterococcus faecalis* and *Enterococcus faecium* from humans in the community, broilers, and pigs in Denmark. Diagn Microbiol Infect Dis; 37, 127–137.
9. Van Wamel WJB, Rooijakkers SHM, Ruyken M, van Kessel KPM, van Strijp JAG (2006) The innate immune modulators staphylococcal complement inhibitor and chemotaxis inhibitory protein of *Staphylococcus aureus* are located on beta-hemolysin-converting bacteriophages. J Bacteriol 188, 1310–1315.
10. Yamaguchi T, Nishifuji K, Sasaki M, Fudaba Y, Aepfelbacher M, Takata T, Ohara M, Komatsuzawa H, Amagai M, Sugai M (2002). Identification of the *Staphylococcus aureus etd* pathogenicity island which encodes a novel exfoliative toxin, ETD, and EDIN-B. Infect Immun; 70, 5835–5845.
11. Jarraud S, Mougel C, Thioulouse J, Lina G, Meugnier H, Forey F, Nesme X, Etienne J, Vandenesch F (2002) Relationships between *Staphylococcus aureus* genetic background, virulence factors, *agr* groups (alleles), and human disease. Infect. Immun. 70, 631–641.
12. Enright MC, Day NPJ, Davies CE, Peacock SJ, Spratt BG (2000) Multilocus sequence typing for characterization of methicillin-resistant and methicillin-susceptible clones of *Staphylococcus aureus*. J Clin Microbiol; 38, 1008–1015.
13. Stegger M, Lindsay JA, Moodley A, Skov R, Broens EM, Guardabassi L (2011) Rapid PCR detection of *Staphylococcus aureus* clonal complex 398 by targeting the restriction-modification system carrying *sau1*-*hsdS1*. J Clin Microbiol; 49, 732–734.
